# Literature and Public Welfare

**Published:** 1920-04-24

**Authors:** 


### THE PUBLIC WELL-BEING.

### LITERATURE AND PUBLIC WELFARE.

### Past and Present Influence.

The influence of literature upon public health—in the large sense of that comprehensive term—has long been a matter of more or less academic discussion. In the days of Shakespeare, to go sufficiently far back for our purpose, practically no attempt was made, either through the drama or in literature, deliberately to call attention to a public nuisance with the idea of impressing it upon the mind and conscience of a nation. In the first place, such traditional civilised legacies as communal stinks, disease and suffering, life at haphazard, steep inequalities between the rich and poor were generally accepted without question, and almost without complaint, as inevitable conditions of human existence, so that writers were not concerned to depict the vile things of life realistically with a view to persuading or hectoring the world into building anew, because, apart from a few monkish chroniclers of the age, they did not realise any more than anybody else that much was amiss. Most of those who preached through the written word confined their energies to the realm of morals and religion, believing that salvation on earth lay through sound doctrine and personal holiness. In the second place, the appeal of the stage and the page was so limited in range that it would have been hopeless to look for any practical or widespreading effect by such methods of oblique propagandism.

Even down to the present time literature, with few exceptions, has played but an insignificant part in bringing about solid amelioration in the conditions of public health. The name of Dickens will at once leap to the tongue in protest. We will certainly give him a place to himself. He wrote extravagantly and inaccurately, he confused bathos with pathos, he was often melodramatic when he intended to be dramatic, he was far from being the ideal type of literary reformer. Yet he captured the attention of a large reading public because undoubtedly he possessed a lively fancy, a keen sense of humour, and a capacity for setting out detail with descriptive point. There were the beginnings of realism in some of his methods, and his treatment of facts had a touch of modern journalism. He was the first of his kind, and in the favourable circumstances of the time, before the full power of the Press had arrived to usurp the influence of the literary author, he was able to arouse effective indignation by his vivid pictures of the foulness of a debtor's goal, the iniquities of small private schools conducted without supervision and without scruple, the brutality and crass ignorance of workhouse administration, and the stupidity and injustice of the law's delays. In all such matters he created an atmosphere which had its ultimate issue in more humane and progressive legislation.

But, after Dickens, who else? Certainly nobody in the same class who exerted anything like an equal influence. Ibsen, perhaps, exerted a temporary influence, or, it might be more correct to say, gained a passing notoriety. Winston Churchill, the aggressive American writer, did something to reform the terrifying conditions in the tinned-meat factories of Chicago, though more by sensational semi-journalistic methods than through exercise of the true functions of literature. One cannot think of any other example of a modern author producing the same consequences in matters of public health that Adam Smith produced, for example, in English political economy or Rousseau in the sweeping developments of French political life. One of the most striking accomplishments in all literature is Balzac's "Country Doctor"; yet this artistic masterpiece, which revealed with consummate power the appalling conditions of rural life in a small French village of the last century—the cretinism, the physical and mental torpor of the inhabitants, the filthy state of the streets and the houses, the ignorance and superstition of all classes, the utter absence of any public spirit in the place, the squalid poverty, the repulsive air of decay that hung over the village like a pall—this tremendous indictment by a great author, what kind of a stir did it make in the arrondissements or the cantons or at the heart of the State? About as much as would be caused by the casting of a pebble into the waters of the Seine. Within the last four or five years, indeed, one has seen villages in Northern France that answered in many particulars to Balzac's unforgettable description.

#### Direct Action.

The truth of the matter is that before Literature (with a capital letter) had broadened the basis of its appeal and assumed its full representative character as an expression of all aspects of humanity, its lowly handmaiden, Journalism, stepped in and changed by "direct action" the face of the world. Let us put the matter in simple terms. An intelligent and observant man seizes upon some defect in the administration of public health or discovers a scandal of sanitation in an urban town. He has no need to wait until he can find somebody to publish a book and embody his discovery in a novel or a treatise. He writes to a newspaper. If his complaint is made good, nine times out of ten it is taken up, "featured" and exposed, and the particular abuse abolished, while in countless instances a public opinion is rapidly made and an instructed Parliament takes action by establishing a new set of principles in legislative reform. That is the common, familiar procedure of the present day. Town-planning, a pure water supply, scientific sewage disposal, first-class hospitals, a highly trained medical service for all—from the very rich to the very poor—healthy

**THE PUBLIC WELL-BEING**—(*continued*).

schools, school clinics, holiday camps, the notification of infectious disease, and a score of other public advantages which are becoming as commonplace as the newspaper itself—these are the consequences of a public opinion formed not by the votaries of Literature but by the humbler representatives of the Press. Literature will always have its own special part to play in moulding opinion. But its action is slow, personal, diffused, and stretches far into the future. The newspaper, because it is a more suitable didactic medium and can immediately concentrate the public interest, will remain the indispensable lever which "gets things done."

